# The combined effects of genetic variation in the *SIRT1* gene and dietary intake of n-3 and n-6 polyunsaturated fatty acids on serum LDL-C and HDL-C levels: a population based study

**DOI:** 10.1186/1476-511X-12-4

**Published:** 2013-01-11

**Authors:** Tomoko Inamori, Toshinao Goda, Nobuhiko Kasezawa, Kimiko Yamakawa-Kobayashi

**Affiliations:** 1 Laboratory of Human Genetics, School of Food and Nutritional Sciences, Graduate School of Integrated Pharmaceutical and Nutritional Sciences, University of Shizuoka, Shizuoka, 422-8526, Japan; 2 Laboratory of Nutritional Physiology, School of Food and Nutritional Sciences, Graduate School of Integrated Pharmaceutical and Nutritional Sciences, University of Shizuoka, Shizuoka, 422-8526, Japan; 3 Department of Data Managements for Health Evaluation & Promotion, Shizuoka Medical Center, Shizuoka, 422-8033, Japan

**Keywords:** LDL-cholesterol, HDL-cholesterol, SIRT1, Fatty acids

## Abstract

**Background:**

Dyslipidemia due to high total cholesterol, LDL-cholesterol, triglycerides, or low HDL-cholesterol is an important risk factor for coronary heart disease (CHD). Both SIRT1 and PUFAs can influence the expression of genes for nuclear receptors and transcription factors related to lipid metabolism such as LXRα, LXRβ, PPARα, SREBP-1c.

**Methods:**

A total of 707 Japanese males and 723 females were randomly selected from the participants who visited a medical center for routine medical check-ups. We analyzed the combined effects of the genotype/haplotype of the *SIRT1* gene and dietary n-6/n-3 PUFA intake ratio on the determination of serum lipid levels.

**Results:**

We found that the *SIRT1* gene marked with haplotype 2 was associated with decreased serum LDL-cholesterol and increased HDL-cholesterol levels. In addition, the associations between the *SIRT1* haplotype 2 and decreased LDL-C and increased HDL-C levels were only observed in the low n-6/n-3 PUFA intake ratio group, but not in the high n-6/n-3 PUFA intake ratio group.

**Conclusions:**

Our findings indicate that the combination of genetic variation in the *SIRT1* gene and dietary n-6 and/or n-3 PUFA intake influence the determination of inter-individual variations of serum levels of LDL-C and HDL-C.

## Background

Dyslipidemia due to high total cholesterol, low density lipoprotein (LDL)-cholesterol, triglycerides, or low high density lipoprotein (HDL)-cholesterol is an important risk factor for coronary heart disease (CHD) [1,2]. Data from family and twin studies suggest that genetic variations account for 40-60% of the inter-individual variations in plasma lipid levels [2,3]. In addition to rare mutations that cause familial dyslipidemia, common genetic variants are considered to significantly contribute to the heritability of plasma lipid levels. For example, genome-wide association studies (GWAS) have reported a growing number of new loci involved in lipid metabolism [4,5]. However, loci identified through GWAS may not fully explain the inter-individual variation in plasma lipid levels.

Sirtuin 1 (SIRT1) belongs to the sirtuin protein family of nicotinamide adenine dinucleotide (NAD^+^)-dependent histone deacetylases conserved in evolution from bacteria to humans [6,7]. Human have seven sirtuin family members, SIRT1-SIRT7, which exhibit with different cellular locations, enzyme activities, target substrates and tissue-specificity. Of these, SIRT1 has been most extensively studied. SIRT1 is a nuclear protein and promotes chromatin silencing and transcriptional repression through histone deacetylation. In addition, more than a dozen non-histone proteins serve as substrates for SIRT1. SIRT1 controls numerous physiological processes and protects cells against stress. A number of studies have shown that SIRT1 orthologs are important mediators of the extension of life span observed from yeast to mammals following calorie restriction. During energy crises such as calorie restriction, NAD^+^ level rise, concomitant with SIRT1 activation [6,7]. Transgenic mice overexpressing SIRT1 have beneficial calorie restriction-like phenotypes, while down-regulation of SIRT1 accelerates the aging phenotype in mice [8].

Furthermore, SIRT1 also has an important function in lipid and glucose metabolism, due to deacetylation of a number of nuclear receptors and transcription factors related to lipid and glucose metabolism such as peroxisome-proliferator activated receptor (PPAR)α, PPARγ, peroxisome proliferator-activated receptor gamma coactivator 1-α (PGC1-α), liver X receptor (LXR)α, LXRβ, forkhead box O (FOXO), AMP-activated protein kinase (AMPK) and sterol response element-binding protein-1c (SREBP-1c) [6-11]. Thus, SIRT1 is associated with lipid metabolism, and variations of the *SIRT1* gene might affect the determination of inter-individual variations of plasma lipid levels.

Fatty acids are no longer just sources of energy, but also fine modulators of cellular signaling and metabolism [12]. There is growing evidence of health benefits of consuming certain types of fats including n-6 and n-3 polyunsaturated fatty acids (PUFAs)[12,13], which are essential fatty acids that are not synthesized de novo by mammals. Several studies have shown that dietary intake of n-6 PUFAs, such as linoleic acid found in vegetable oils, may reduce CHD risk by beneficial effects on serum total cholesterol, LDL cholesterol, and insulin sensitivity [14], while n-3 PUFA derived from fish has also been shown to decrease serum triglyceride and increase HDL-cholesterol, which is associated with more efficient reverse cholesterol transport and a reduced risk of CHD [15,16]. It is also reported that n-3 PUFA have anti-inflammatory effects [16]. However, the cellular mechanisms underling the beneficial effects of n-6 and n-3 PUFA on lipid profile and CHD prevention are not completely understood. Recently, n-6 and n-3 PUFAs were shown to be critical for modulation of expression in several nuclear receptors and transcription factors, including LXRα, LXRβ, PPARα, SREBP-1c, hepatocyte nuclear factor (HNF)-4α, and nuclear factor-κB (NFκB) [17]. The majority of these genes play key roles in lipid metabolism, and their expression is also modulated by SIRT1 [6-11]. Furthermore, it was reported that calorie restriction and dietary n-3 PUFA intake induce the similar beneficial effects such as anti-inflammation, preventing obesity and increasing expression insulin sensitivity in mice [18]. Recently, it was shown that the anti-inflammatory effect of n-3 PUFAs might be mediated through activation of AMPK/SIRT1 pathways; because of n-3 PUFAs increased expression, phosphorylation and activity of AMPK in macrophages, which further leaded to SIRT1 over-expression [19]. These data indicate the possibility that dietary n-3 PUFAs intake modify the SIRT1 activity *in vivo.*

In the present study, we investigated whether the common variations in the *SIRT1* gene are potential contributors to inter-individual variations in serum lipid levels. Furthermore, we analyzed the interaction of the common *SIRT1* variants and dietary n-3 and n-6 PUFAs intake on determination of serum lipid levels.

## Results

Table 1 shows demographic and biochemical characteristics, and dietary intake of our subjects. There was a significant difference in these data between male and female. As such, we analyzed the data separately in male and female. We examined the relationships between the genotypes/haplotypes of the three single nucleotide polymorphisms (SNPs) (rs7069102, rs2273773, rs3818292) in the *SIRT1* gene and metabolic phenotypes such as levels of fasting serum total cholesterol, LDL-cholesterol, HDL-cholesterol, triglycerides, glucose, hemoglobin A1c (HbA1c), and body mass index (BMI). These three SNPs were in linkage disequilibrium (LD) with each other (|D’| > 0.87), and we constructed a haplotype using these three SNPs. There were three common haplotypes with frequencies of >16%, which accounted for 98% of all chromosomes in our subjects (see Additional file 1: Table S1, and Table S2).

**Table 1:** Characteristics of the study subjects

	**Male**	**Female**	**P-value**
n	707	723	
Age (years)	53.8 ± 5.2	53.0 ± 5.1	0.075
BMI (kg/m^2^)	23.5 ± 2.9	22.2 ± 3.1	<0.0001
Total Cholesterol (mg/dl)	209.9 ± 31.4	217.7 ± 31.4	<0.0001
LDL-Cholesterol (mg/dl)	130.4 ± 29.8	131.9 ± 30.0	0.38
HDL-Cholesterol (mg/dl)	57.0 ± 16.3	71.5 ± 17.1	<0.0001
Triglyceride (mg/dl)	137.8 ± 102.5	86.8 ± 45.8	<0.0001*
glucose (mg/dl)	99.5 ± 15.4	92.7 ± 10.9	<0.0001*
HbA1c (%)	5.3 ± 0.59	5.1 ± 0.44	<0.0001
Smokers (%)	42.5	8.4	<0.0001
**Dietary intakes**			
Energy (kcal/day)	2106.5 ± 567.0	1615.5 ± 414.9	<0.0001
Total fat (%energy)	25.9 ± 6.0	28.2 ± 5.7	<0.0001
Carbohydrate (%energy)	57.1 ± 7.8	56.0 ± 7.0	0.012
SFA (%energy)	6.5 ± 1.8	7.6 ± 2.0	<0.0001
PUFA (%energy)	6.7 ± 1.7	7.0 ± 1.6	0.0019
n-6 PUFA (%energy)	5.4 ± 1.5	5.7 ± 1.4	0.0004
n-3 PUFA (%energy)	1.5 ± 0.49	1.5 ± 0.44	0.42
Alcohol (g/day)	28.7 ± 31.6	6.5 ± 14.6	<0.0001

*BMI* body mass index, *HbA1c* hemoglobin A1c, *SFA* saturated fatty acid, *PUFA* polyunsaturated fatty acid.

Data are expressed as mean ± SD or percentage.

P-values were calculated by t-test or χ2 test.

The subject numbers whose data for LDL-Cholesterol, HbA1c, and dietary intake available are 1430, 1408, and 1248, respectively.

*Statistical tests for triglyceride and glucose levels were calculated on log-transformed values.

In males, significant associations were observed between LDL-cholesterol level and all three SNPs or haplotype 2, and between HDL-cholesterol level and haplotype 2. In females, significant associations were observed between LDL-cholesterol level and haplotype 2, and between HDL-cholesterol levels and SNP (rs3818292) or haplotype 1. The other metabolic traits including serum total cholesterol, glucose, HbA1c levels, and BMI were not associated with the genotype/haplotype of the *SIRT1* gene (Table 2).

**Table 2:** Relationships between genotypes/haplotypes of the SIRT1 gene and metabolic phenotypes

	**Genotype**		**Total-Cholesterol**		**LDL-Cholesterol**		**HDL-Cholesterol**		Triglycerides*		Glucose*		**HbA1c**		BMI**
		**n**	**(mg/dl)**	**n**	**(mg/dl)**	**n**	**(mg/dl)**	**n**	**(mg/dl)**	**n**	**(mg/dl)**	**n**	**(%)**	**n**	**(kg/m**^**2**^**)**
**Male**															
rs7069102	CC	487	208.9 ± 31.4	482	129.4 ± 29.1	487	57.5 ± 16.7	487	135.2 ± 105.4	487	99.7 ± 15.7	484	5.27 ± 0.60	487	23.5 ± 2.8
(intron 4)	CG	207	212.7 ± 31.4	203	133.5 ± 31.0	207	55.5 ± 15.3	207	146.0 ± 97.3	207	99.2 ± 14.8	206	5.29 ± 0.58	207	23.4 ± 2.9
	GG	13	203.8 ± 31.8	13	122.7 ± 34.3	13	60.6 ± 13.7	13	100.8 ± 55.5	13	93.5 ± 12.1	13	5.33 ± 0.61	13	24.1 ± 7.1
	P value		0.067		**0.010**		0.42		**0.017**		0.31		0.46		0.55
rs2273773	TT	315	212.4 ± 33.2	311	134.4 ± 30.6	315	55.3 ± 15.7	315	136.4 ± 103.1	315	99.2 ± 14.8	313	5.29 ± 0.58	315	23.4 ± 2.9
(exon 5)	TC	294	208.8 ± 29.6	290	127.1 ± 28.5	294	58.1 ± 16.4	294	140.2 ± 106.8	294	99.8 ± 16.9	293	5.25 ± 0.64	294	23.5 ± 2.9
	CC	98	207.8 ± 30.4	97	127.7 ± 30.0	98	58.9 ± 17.5	98	134.7 ± 87.3	98	99.5 ± 12.2	97	5.31 ± 0.47	98	23.7 ± 2.8
	P value		0.32		**0.024**		0.054		0.86		0.71		0.81		0.40
rs3818292	AA	313	212.4 ± 33.4	309	134.3 ± 30.6	313	55.4 ± 15.6	313	136.1 ± 103.7	313	99.4 ± 14.8	311	5.29 ± 0.59	313	23.4 ± 2.9
(intron 5)	AG	297	208.0 ± 29.4	293	127.3 ± 28.4	297	57.9 ± 16.4	297	140.6 ± 106.0	297	99.7 ± 16.9	296	5.26 ± 0.64	297	23.5 ± 3.0
	GG	97	207.8 ± 30.7	96	127.7 ± 30.2	97	59.1 ± 17.8	97	134.4 ± 87.7	97	99.0 ± 12.0	96	5.31 ± 0.46	97	23.7 ± 2.7
	P value		0.27		**0.024**		0.10		0.70		0.75		0.85		0.34
haplotype 1	+	515	210.6 ± 32.4	510	131.6 ± 30.1	515	56.6 ± 16.2	515	135.7 ± 102.2	515	99.9 ± 16.5	513	5.28 ± 0.63	515	23.5 ± 2.8
(C-T-A)	-	192	208.1 ± 28.6	188	127.2 ± 28.7	192	58.0 ±16.6	192	143.2 ± 103.5	192	98.2 ± 11.6	190	5.28 ± 0.48	192	23.5 ± 3.2
	P value		0.53		0.150		0.24		0.21		0.34		0.59		0.86
haplotype 2	+	384	208.0 ± 29.7	379	127.4 ± 28.8	384	58.1 ± 16.7	384	139.8 ± 102.8	384	99.7 ± 16.0	382	5.27 ± 0.60	384	23.5 ± 2.7
(C-C-G)	-	323	212.2 ± 33.2	319	134.1 ± 30.5	323	55.6 ± 15.7	323	135.3 ± 102.3	323	99.2 ± 14.7	321	5.29 ± 0.58	323	23.5 ± 3.2
	P value		0.17		**0.011**		**0.049**		0.47		0.60		0.68		0.14
haplotype 3	+	213	212.5 ± 31.6	209	133.2 ± 31.3	213	55.5 ± 14.9	231	144.4 ± 97.1	213	99.1 ± 14.8	212	5.30 ± 0.59	213	22.4 ± 3.2
(G-T-A)	-	494	208.8 ± 31.3	489	129.2 ± 29.1	494	57.6 ± 16.8	494	134.9 ± 104.8	494	99.6 ± 15.6	491	5.27 ± 0.59	494	23.5 ± 2.8
	P value		0.069		0.055		0.31		0.053		0.57		0.37		0.51
**Female**															
rs7069102	CC	504	217.7 ± 31.9	492	131.7 ± 30.9	504	71.6 ± 17.2	504	85.7 ± 43.2	504	92.9 ± 11.3	493	5.14 ± 0.45	504	22.2 ± 3.2
(intron 4)	CG	198	217.9 ± 30.2	190	132.0 ± 28.0	198	71.8 ± 17.0	198	87.8 ± 50.2	198	92.0 ± 9.0	192	5.16 ± 0.40	198	22.1 ± 2.9
	GG	21	215.0 ± 31.1	20	134.4 ± 26.3	21	63.6 ± 15.9	21	102.8 ± 59.6	21	94.3 ± 17.7	20	5.11 ± 0.44	21	22.7 ± 2.8
	P value		0.63		0.82		0.12		0.13		0.75		0.20		0.50
rs2273773	TT	310	218.6 ± 32.0	300	134.0 ±30.8	310	70.0 ± 16.8	310	89.9 ± 49.3	310	93.3 ± 12.9	301	5.17 ± 0.50	310	22.2 ± 3.0
(exon 5)	TC	320	215.6 ± 31.0	315	129.0 ± 29.7	320	72.5 ± 17.2	320	83.5 ± 40.8	320	91.8 ± 8.9	316	5.12 ± 0.37	320	22.1 ± 3.1
	CC	93	221.7 ± 30.4	87	135.0 ± 27.3	93	72.8 ± 17.7	93	87.6 ± 49.4	93	93.4 ±10.0	88	5.15 ± 0.44	93	22.6 ± 3.4
	P value		0.23		0.091		0.096		0.25		0.14		0.70		0.66
rs3818292	AA	303	219.1 ± 31.9	294	134.4 ± 30.4	303	69.8 ± 16.9	303	90.3 ± 49.1	303	93.5 ± 13.0	295	5.17 ± 0.50	303	22.2 ± 3.0
(intron 5)	AG	309	214.9 ± 30.8	305	128.7 ± 30.1	309	72.4 ± 17.0	309	83.3 ± 41.4	309	91.7 ± 9.1	305	5.12 ± 0.37	309	22.1 ± 3.2
	GG	111	221.8 ± 30.9	103	133.8 ± 27.9	111	73.4 ± 17.8	111	86.8 ± 47.6	111	93.2 ± 9.2	105	5.13 ± 0.42	111	22.5 ± 3.30
	P value		0.11		0.061		**0.047**		0.17		0.090		0.94		0.83
haplotype 1	+	515	216.9 ± 31.8	504	131.7 ± 31.1	515	70.8 ± 16.8	515	86.6 ± 44.2	515	92.7 ± 11.1	505	5.14 ± 0.45	515	22.1 ± 3.1
(C-T-A)	-	208	219.7 ± 30.1	198	132.2 ± 26.9	208	73.2 ± 17.7	208	87.2 ± 49.6	208	92.7 ± 10.5	200	5.14 ± 0.41	208	22.3 ± 3.1
	P value		0.47		0.77		**0.045**		0.65		0.70		0.43		0.88
haplotype 2	+	408	216.9 ± 31.0	397	130.1 ± 29.4	408	72.6 ± 17.2	408	84.5 ± 43.0	408	92.1 ± 9.1	399	5.13 ± 0.38	408	22.2 ± 3.2
(C-C-G)	-	315	218.7 ± 31.9	305	134.1 ± 30.6	315	69.9 ± 16.9	315	89.7 ± 49.0	315	93.4 ± 12.9	306	5.16 ± 0.50	315	22.2 ± 3.0
	P value		0.27		**0.039**		0.065		0.16		0.088		0.85		0.95
haplotype 3	+	216	217.5 ± 30.4	208	132.4 ± 28.0	216	71.0 ± 17.1	216	88.9 ± 51.5	216	92.2 ± 10.2	209	5.15 ± 0.41	216	22.2 ± 2.9
(G-T-A)	-	507	217.8 ± 31.8	494	131.6 ± 30.8	507	71.7 ± 17.1	507	85.8 ± 43.1	507	92.9 ± 11.2	496	5.14 ± 0.45	507	22.2 ± 3.2
	P value		0.96		0.67		0.47		0.22		0.92		0.29		0.72

Values are shown as mean ± SD.

P-values were calculated by multiple linear regression analyses incorporating age, BMI, current smoking and alcohol intake as covariates.

*Statistical tests for glucose and TG levels were calculated on log-transformed values.

**P-values for BMI were calculated by multiple linear regression analyses incorporating age, current smoking and alcohol intake as covariates.

Statistically significant P-values (P < 0.05) are indicated by bold.

The carriers of haplotype 2 had lower serum LDL-C and higher HDL-C levels than for the non-carriers of haplotype 2, although in females the relationship between HDL-C levels and haplotype 2 was not statistically significant (P = 0.065). These data indicate that haplotype 2 is a beneficial haplotype associated with decreased LDL-cholesterol and increased HDL-cholesterol levels.

Next, to examine whether dietary n-6 and n-3 PUFA intake modulates the association between *SIRT1* haplotypes and LDL-C and/or HDL-C levels, we classified subjects into two subgroups based on the population median of dietary n-6/n-3 PUFA intake ratio (3.79 for males, 3.93 for females). Significant associations between *SIRT1* haplotype 2 and LDL-C and/or HDL-C levels were observed in only the group with a low n-6/n-3 PUFA intake ratio, but were not observed in the group with a high n-6/n-3 PUFA intake ratio (Table 3). In the group with a low n-6/n-3 PUFA intake ratio, the association between haplotype 3 and LDL-C levels in male (P = 0.033), and that between haplotype 1 and HDL-C levels in female were also observed (P = 0.022).

**Table 3:** The combined effects of SIRT1 haplotype and n-6/n-3 PUFA intake ratio on serum LDL-C and HDL-C levels

	**Male**				**Female**			
	**Low n6/n3 intake group(<3.79)**		**High n6/n3 intake group(≧3.79)**		**Low n6/n3 intake group(<3.93)**		**High n6/n3 intake group(≧3.93)**	
	**n**	**(mg/dl)**	**n**	**(mg/dl)**	**n**	**(mg/dl)**	**n**	**(mg/dl)**
**LDL-Cholesterol**								
Haplotype 1 (+)	221	133.5 ± 31.1	232	131.1 ± 29.3	215	133.6 ± 30.5	213	131.2 ± 31.3
Haplotype 1 (−)	88	127.0 ± 29.8	74	129.4 ± 28.6	93	131.3 ± 26.2	84	131.1 ± 28.1
P-value		0.13		0.68		0.53		0.87
Haplotype 2 (+)	179	128.0 ± 30.5	149	129.4 ± 28.6	187	129.7 ± 27.6	163	130.2 ± 30.6
Haplotype 2 (−)	130	136.6 ± 30.7	157	131.9 ± 29.7	121	137.9 ± 31.0	134	132.4 ± 30.1
P-value		**0.0089**		0.28		**0.0085**		0.68
Haplotype 3 (+)	82	136.8 ± 30.2	95	131.5 ± 31.6	82	131.1 ± 25.1	98	134.0 ± 30.2
Haplotype 3 (−)	227	129.8 ± 30.9	211	130.3 ± 28.1	226	133.6 ± 30.6	199	129.8 ± 30.5
P-value		**0.033**		0.56		0.99		0.49
**HDL-Cholesterol**								
Haplotype 1 (+)	223	55.7 ± 16.8	234	56.8 ± 15.9	218	72.1 ± 17.2	220	69.0 ± 16.7
Haplotype 1 (−)	89	58.3 ± 16.7	77	57.0 ± 16.0	96	76.9 ± 17.8	91	69.5 ± 17.4
P-value		0.16		0.72		**0.022**		0.92
Haplotype 2 (+)	181	57.6 ± 17.1	152	57.4 ± 16.4	190	75.5 ± 17.4	170	69.4 ± 17.1
Haplotype 2 (−)	131	54.8 ± 16.4	159	56.4 ± 15.4	124	70.5 ± 17.4	141	68.9 ± 16.7
P-value		0.079		0.27		**0.032**		0.83
Haplotype 3 (+)	84	53.8 ± 14.9	97	56.6 ± 15.0	84	71.3 ± 17.1	104	69.6 ± 17.7
Haplotype 3 (−)	228	57.4 ± 17.4	214	57.0 ± 16.3	230	74.4 ± 17.6	207	68.9 ± 16.5
P-value		0.14		0.90		0.15		0.72

Values are shown as mean ± SD.

P-values were calculated by multiple linear regression analyses incorporating age, BMI, current smoking and alcohol intake as covariates.

Statistically significant P-values (P < 0.05) are indicated by bold.

These findings indicate that the combination of genetic variations in the *SIRT1* gene and dietary n-6 and/or n-3 PUFA intake influence the determination of inter-individual variations of serum levels of LDL-C and HDL-C.

## Discussion

Plasma levels of high LDL-C and low HDL-C are considered major determinants of susceptibility to CHD in the general population. The determination of plasma lipid levels is controlled by multiple pathways and influenced by complex interactions between many different genes and environmental factors such as diet intake.

In the present study, we found that the combination between the *SIRT1* gene marked with haplotype 2 and low n-6/n-3 PUFA intake ratio was related to beneficial effects on serum lipid profile, a decreased LDL-C and an increased HDL-C levels, in both males and females. Both SIRT1 and PUFAs can influence the expression of genes for nuclear receptors and transcription factors related to lipid metabolism including LXRα, LXRβ, PPARα, SREBP-1c [9-11,17]. Recently, it was reported that n-3 PUFAs activate AMPK/SIRT1 pathways in macrophages [19]. These data indicate the possibility that the activity or function of SIRT1 might be modified by dietary n-3 PUFAs intake. In addition, it remains possible that the activity of SIRT1 marked with haplotype 2 is more easily increased by n-3 PUFA intake without haplotype 2. At present, we have no direct evidence that the activity of SIRT1 was influenced by dietary n-3 PUFA intake, and the *SIRT1* gene marked with haplotype 2 in this study caused alterations in the activity and/or function of SIRT1 deacetylase.

Genetic variants of the *SIRT1* gene have been shown to be associated with human diabetes and obesity-related phenotypes in several previous studies [20-22], while only a few genetic association studies for the *SIRT1* gene and lipid metabolism have been reported [23]. Almost all genetic variants detected in the *SIRT1* gene were synonymous, only a few variants with possible functional changes were reported in the promoter region [24]. The three SNPs that we analyzed in this study are also non-functional variants. And we could not detect associations between genotype/haplotype of the *SIRT1* gene and diabetes and obesity-related phenotypes including serum glucose levels, HbA1c, and BMI in this study (Table 2). However, it remains possible that the *SIRT1* gene marked with haplotype 2 might exist in linkage disequilibrium with other new functional variants.

Further *in vivo* and *in vitro* studies are needed to assess the role for dietary n-3 or n-6 PUFA intake and genetic variation marked with haplotype 2 in the *SIRT1* gene on changes in SIRT1 expression or function.

The lower saturated fat (meat) and higher n-3-PUFA (fish) in the Japanese diet was suggested to contribute to the lower prevalence of hypercholesterolemia and lower risk of CHD [25]. Furthermore, the beneficial *SIRT1* variant marked with haplotype 2 is rather common in the Japanese population (the frequency of haplotype 2 is 0.33). Future studies are required to ascertain whether the combination between the *SIRT1* gene marked with haplotype 2 and low n-6/n-3 PUFA intake ratio can produce beneficial effects on serum lipid profile in other populations containing young people, children, or other people with different dietary habits. Prospective cohort studies are also required to determine the interactions between genetic variations of the *SIRT1* gene and dietary PUFA on serum lipid profile. It is important to determine the potential for interactions between genetic and modifiable environmental factors such as dietary nutrient intake to establish a preventive method for common diseases such as dyslipidemia and CHD.

## Conclusion

We found that the genotype/haplotype of the *SIRT1* gene is associated with serum LDL-C and HDL-C levels, and that the variant marked with haplotype 2 is associated with decreased LDL-cholesterol and increased HDL-cholesterol levels. In addition, the associations between the *SIRT1* haplotype 2 and decreased LDL-C and/or increased HDL-C levels were only observed in the low n-6/n-3 PUFA intake ratio group, but not in the high n-6/n-3 PUFA intake ratio group. These findings indicate that the combination of genetic variations in the *SIRT1* gene and dietary n-6 and/or n-3 PUFA intake can influence the determination of inter-individual variations of serum levels of LDL-C and HDL-C. An understanding of the interactions of genetic and environmental factors on the prevalence of dyslipidemia is useful for prediction and prevention of CHD, as high LDL-C and low HDL-C levels are important risk factors for CHD.

## Subjects and methods

### Subjects

A total of 707 Japanese males and 723 females were randomly selected from participants who visited a medical center near the University of Shizuoka for routine medical check-ups. Subjects were all Japanese, and ranged from 45–65 years (mean age, 53.8 ± 5.2 males, 53.0 ± 5.1 females). People taking medication for dyslipidemia and/or diabetes were excluded from the study subjects. After overnight fasting, blood samples were collected from each subject. Written informed consent was obtained from all subjects and this study was approved by the Ethics Committee of the University of Shizuoka.

### DNA analysis

Genomic DNA was isolated from peripheral leucocytes by the phenol extraction method. We analyzed the genotypes and haplotypes of three tag SNPs (rs7069102 [intron 4], rs2273773 [exon 5, Leu27Leu], rs3818292 [intron 5]) in the *SIRT1* gene, which were selected from the HapMap database (http://hapmap.ncbi.nlm.nih.gov/index.html.en), based on their minor allele frequencies (MAF) in the Japanese population and previous reports describing the *SIRT1* polymorphism [20,23]. The *SIRT1* gene is localized to chromosome region 10q21.3 with 9 exons. To date, approximately 100 common SNPs (MAF≧ 0.05) have been on the dbSNP database (http://www.ncbi.nlm.nih.gov/snp?term). The majority of SNPs exist in intron or untranslated regions.

The genotypes of these three SNPs were determined for each subject using the PCR-restriction fragment length polymorphism method. The haplotypes and their frequencies were estimated by the maximum-likelihood method with an expectation-maximization-based algorithm using the SNPAlyze program (Dynacom, Tokyo, Japan).

### Dietary assessment

Dietary intake was assessed using a brief-type self-administered diet history questionnaire (BDHQ). The BDHQ was developed based on the self-administered diet history questionnaire (DHQ), which had been validated using three different standard methods for dietary assessment [26,27]. The BDHQ were designed to obtain dietary habits for the previous month from validating dietary intake for 58 food and beverage items which are commonly consumed in general Japan populations [26,27]. With respect to n-3 and n-6 PUFA, the majority food sources are α-linolenic acid (vegetable oil), EPA (fish and shellfish), DHA (fish and shellfish) and linoleic acid (vegetable oil)*,* arachidonic acid (fish, organ meats and egg), respectively. Intake of fat, carbohydrate, protein, and fatty acids was expressed as percentages of the total non-alcohol energy intake. Dietary data were available for 1248 subjects (87.3%) in this study.

### Statistical analyses

The relationships between genotypes/haplotypes of the three SNPs of the *SIRT1* gene and metabolic parameters including serum lipid and glucose levels were analyzed by multiple linear regression analyses incorporating age, BMI, alcohol intake, and smoking status as covariates. Statistical analyses were performed using the JMP 9 software package (SAS Institute, Cary, NC, USA). The coefficients of linkage disequilibrium (LD) value (|D’| and r^2^) among three SNPs were calculated by using the SNPAlyze program (Dynacom, Tokyo, Japan).

## Abbreviations

LDL: Low density lipoprotein; HDL: High density lipoprotein; CHD: Coronary heart disease; GWAS: Genome-wide association study; SIRT1: Sirtuin 1; PPAR: Peroxisome-proliferator activated receptor; PGC-1α: Peroxisome proliferator-activated receptor gamma coactivator 1-alpha, LXR, Liver X receptor; FOXO: Forkhead box O; AMPK: AMP-activated protein kinase; SREBP: Sterol response element-binding protein; PUFA: Polyunsaturated fatty acid; SNP: Single nucleotide polymorphism; HbA1c: Hemoglobin A1c; BMI: Body mass index; LD: Linkage disequilibrium.

## Competing interests

The authors declare that they have no competing interests.

## Authors’ contributions

TI and KYM managed the study and carried out the genetic analysis, drafting the manuscript. TG carried out the dietary assessment. NK collected study subjects. All authors read and approved the final manuscript.

## Supplementary Material

Additional file 1 **Table1.** The pair wise linkage disequilibrium (LD) values of |D'| (upper) and r^2^ (lower). **Table 2.** Estimated *SIRT1* haplotypes and frequencies in the Japanese population. Click here for file
