# Supplementary material for: The combined effects of genetic variation in the SIRT1 gene and dietary intake of n-3 and n-6 polyunsaturated fatty acids on serum LDL-C and HDL-C levels: a population based study
Source: Lipids Health Dis. 2013 Jan 11;12:4. doi: 10.1186/1476-511X-12-4 (PMC3554548; doi:10.1186/1476-511X-12-4)
Supplement: Additional file 1 — Table1. The pair wise linkage disequilibrium (LD) values of |D'| (upper) and r2 (lower). Table 2. Estimated SIRT1 haplotypes and frequencies in the Japanese population. [file 1476-511X-12-4-S1.pdf]

Additional Table1 The pair wise linkage disequilibrium (LD) values of |D'| (upper) and $r^2$ (lower)

| | \|D'\| | | |
|---|---|---|---|
| $r^2$ | rs7069102 | rs2273773 | rs3818292 |
| rs7069102 | | 0.95 | 0.87 |
| rs2273773 | 0.095 | | 0.97 |
| rs3818292 | 0.082 | 0.92 | |

Additional Table 2 Estimated SIRT1 haplotypes and frequencies in the Japanese population

| | rs7069102 | rs2273773 | rs3818292 | Frequencies (%) |
|---|---|---|---|---|
| Haplotype 1 | C | T | A | 48.4 |
| Haplotype 2 | C | C | G | 33.4 |
| Haplotype 3 | G | T | A | 16.1 |
